# Study of Treatment Methods for Surrounding Soft Tissues of Implants Following Mandibular Reconstructions with Fibula-Free Flaps

**DOI:** 10.3390/dj8030067

**Published:** 2020-07-03

**Authors:** Kazuhiko Nakaue, Satoshi Horita, Mitsuhiko Imada, Kazuhiko Yamamoto, Tsutomu Sugiura, Nobuhiro Ueda, Nobuhiro Yamakawa, Yohei Nakayama, Tadaaki Kirita

**Affiliations:** 1 Department of Oral and Maxillofacial Surgery, Nara Medical University, 840 Shijo-cho, Kashihara 634-8521, Japan; horisato@naramed-u.ac.jp (S.H.); mitsuhiko-imada@naramed-u.ac.jp (M.I.); kazuyama@naramed-u.ac.jp (K.Y.); sugiurat@naramed-u.ac.jp (T.S.); n-ueda@naramed-u.ac.jp (N.U.); yamanobu@naramed-u.ac.jp (N.Y.); yohei-323@naramed-u.ac.jp (Y.N.); tkirita@naramed-u.ac.jp (T.K.); 2 Department of Oral and Maxillofacial Surgery, Nara Prefecture General Medical Center, 897-5 Shitijounisimati, Nara 630-8581, Japan; 3 Department of Oral Surgery, Minami-nara General Medical Center 8-1, Fukugami, Oyodo, Yoshino, Nara 638-8551, Japan

**Keywords:** free gingival graft (FGG), maxillofacial reconstruction, keratinized mucosa, soft tissue treatment, fibula-free flap mandible reconstruction

## Abstract

In maxillofacial reconstruction implant treatment, unsatisfactory soft tissue treatment of the area around the implant may lead to inflammation. As a result, appropriate soft tissue treatment is critical. To the best of our knowledge, there are no studies that compare the different tissue treatment methods available. Hence, in this study, we compare three soft tissue treatment methods around implants after mandibular reconstruction is achieved with a fibula-free flap. Out of 33 patients who underwent mandible reconstruction using fibula-free flaps between 2006 and 2015, 5 were selected for this study. A total of 17 implants were used for treatment by the final prosthetics of the five patients. Three soft tissue treatment methods with free gingival graft (FGG) were evaluated, namely, installing a splint in a modified abutment to protect the wounded area during a palatal mucosa transplant (method 1), installing a splint or dentures to a locator abutment (method 2), and the use of screw-in fixed dentures (method 3). The method that could guarantee the widest keratinized mucosa was the screw-in fixed denture method. The results of our study indicated that employing screw-in fixed dentures for FGG may be a useful soft tissue treatment for mandible reconstruction implants.

## 1. Introduction

The successful application of implant treatments in patients undergoing fibula-free flap mandibular reconstruction has been proven to be a useful technique, and this is well documented in the scientific literature [1,2,3,4]. However, the fibula-free flap is a skin appendage typically composed of fat, muscles and thick hair. Therefore, its use as soft tissue around implants is not suitable, as it may induce inflammation [2,5]. Some attempts have been made to use alternative soft tissues around implants. Most of these attempts have focused on the use of free gingival grafts (FGG) to obtain a thin layer of soft tissue around implants.

The formation of a minimal-width keratinized mucosa in ordinary implant surgeries has been the subject of intense investigation in the scientific literature. Warrer et al. [6] reported, in a study involving monkeys, that when plaque is deposited around implants, little tissue damage and bone loss occurs if keratinized mucosa is present. Listgarten et al. [7] reported that inserting implants in movable tissue results in an interface at the junctional epithelium. This interface can be easily destroyed, and is susceptible to inflammation. To avoid these complications, researchers have suggested inserting implants in keratinized mucosa. Matsui [8] stated that at least 5 mm of keratinized mucosa in the area around the implant is required, because the tissue around an implant exhibits weak resistance when compared to that around natural teeth. In contrast, Cairo et al. [9] conducted a systematic review that determined, from a statistical analysis, that keratinized mucosa does not affect implant prognosis. Lin et al. [10] observed that the absence of an adequate keratinized mucosa around dental implants is associated with increased plaque accumulation, tissue inflammation, mucosal recession and attachment loss.

Other widely investigated methods are as follows: (1) Sub-periosteal dissection with denture-guided epithelial regeneration (SD-DGER) [2,11]. This method promotes mucosa formation via dentures, after the removal of the skin flap. (2) Skin flap removal only [5]. (3) FGG, after the removal of the skin flap [2,10]. (4) The dermabrasion technique [12]. Further, this approach can be used as a soft tissue treatment after mandibular reconstruction. Moreover, Chang et al. [10] reported significantly less bone loss in a group of fibula-free flap mandibular reconstruction patients subjected to FGG for implant treatment, as compared with those who were subjected only to skin flap reduction. Meanwhile, Sozzi et al. [5] obtained a larger keratinized mucosa width with SD-DGER than with flap removal and FGG.

In FGG, it is common practice to reposition a mobile mucosa to the root tip and to sew the graft membrane onto the gingival gums, which are kept immobile and are protected with a periodontal dressing. However, complications occur with mucosa grafts in mandibular reconstruction with a fibula-free flap, because they must be grafted not only on the buccal side, but all around the implant. Additionally, a variety of conditions must first be met, because the graft is wide, the alveolar ridge is low, and there is insufficient tissue around the implant to fix a mucosal graft. Therefore, we considered that Sozzi et al. [5] obtained a large keratinized mucosa width with SD-DGER, as compared to flap removal and FGG, and that FGG was useful in the study conducted by Chang et al. [10] However, to the best of our knowledge, there are no consistent treatment plans or comparisons between the various surgical techniques used in the literature. Therefore, the present study aimed to compare three soft tissue treatment methods, using FGG, for implants after mandibular reconstruction with fibula-free flap. Additionally, the width of the keratinized mucosa obtained around the implant was measured, and compared among the different treatment methods.

## 2. Materials and Methods

**Figure 1:**
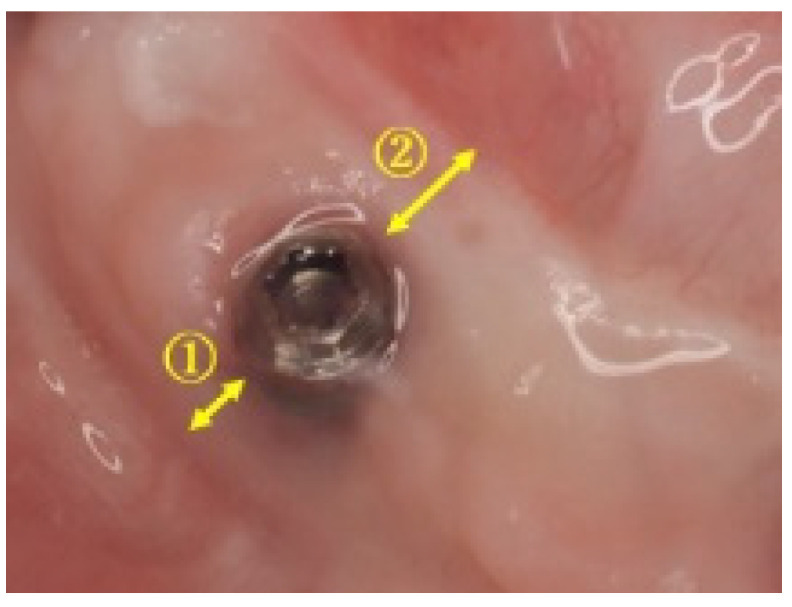
Measurements of keratinized mucosa width (Case 3): keratinized mucosa width was measured on the buccal side ① and the lingual side ② of the implant.

Among the 33 patients who underwent mandibular reconstruction using fibula flaps from 2006 to 2015, 5 patients who had undergone implant treatment and been fitted the final prosthesis were selected for this study. A total of 17 implants were used for the five patients. For four implants in Case 4, a different soft tissue treatment was performed, because a suitable keratinized mucosa width could not be obtained. However, each of the soft tissue treatments was evaluated in all patients. The width of the keratinized mucosa obtained was measured on both the buccal and lingual sides of the implant, three months after FGG surgery (Figure 1). The Mann–Whitney U test was used for statistical analysis. This study was approved by the Nara Medical University Ethics Committee on 5 November 2019 (approval number 2430).

Excluding one patient who received mandibular reconstruction for osteoradionecrosis, none of the patients were subjected to radiation treatment. In four of the five patients, skin flap reduction and alveolar bone graft surgery were performed; this was followed by primary/secondary implant surgeries and soft tissue treatment. For the skin flap reduction and alveolar bone graft surgery, the largest possible quantity of fat and other unwanted tissue was removed from the skin flaps, and the fibula was sewn into the lingual and buccal periosteum, forming the alveolar ridge. In addition, there was not enough clearance in one patient; in this case, the fibula was ground down to ensure enough clearance. Two of the other four patients had undergone soft tissue treatment at the same time as their secondary implant surgery. Case 5 required treatment of the entire jaw; therefore, after installing the implants, skin flap reduction, alveolar bone graft surgery and FGG were performed alongside the secondary implant surgery (Table 1). Only Brånemark System Mk III implants (Nobel Biocare, Kloten Sweden) were used in this study.

**Figure 2:**
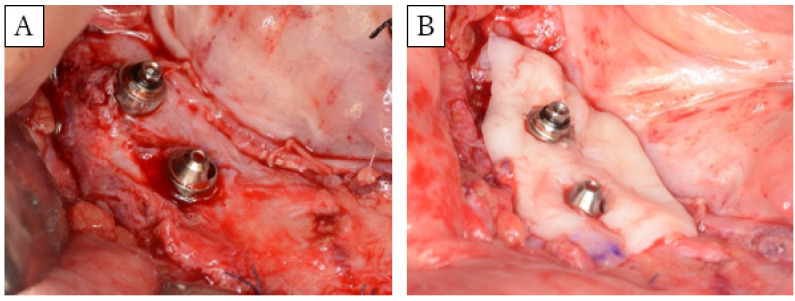
Images of FGG surgery. (**A**) Formation of a graft bed in a 10-mm region around the implant. (**B**) Grafting palatal mucosa to cover a 5-mm radius around the implant.

FGGs, of split-thickness skin, were conducted in four of the five patients. Skin flaps were removed from the fibula and fixed to the lingual and buccal periosteum, forming roughly a 10-mm graft bed in the area around the implant (Figure 2A). Palatal mucosa was grafted to an area of approximately 5 mm surrounding the implant (Figure 2B). The wounded area was protected using the following methods: Method 1, a splint installed in a modified abutment; Method 2, a splint (or dentures) fixed by a locator abutment dressed with a COE-PAK^®^ (YOSHIDA, Japan); and Method 3, denture rebasing conducted during surgery, in which a temporary abutment was installed and dentures were fixed in with screws. The wounded area was protected for a period of four weeks in Method 1. For Method 2, the dentures (splint) were removed after two weeks, the wounded area and dentures were washed, and COE-PAK^®^ used again to protect the wounded area; the protection was discontinued after two weeks. For Method 3, the dentures were removed after four weeks, the wounded area was washed, and the dentures were adjusted. The same treatment was conducted weekly, after which a transition to dentures was achieved using a locator abutment.

## 3. Representative Cases

### 3.1. Case 2 (Method 1)

**Figure 3:**
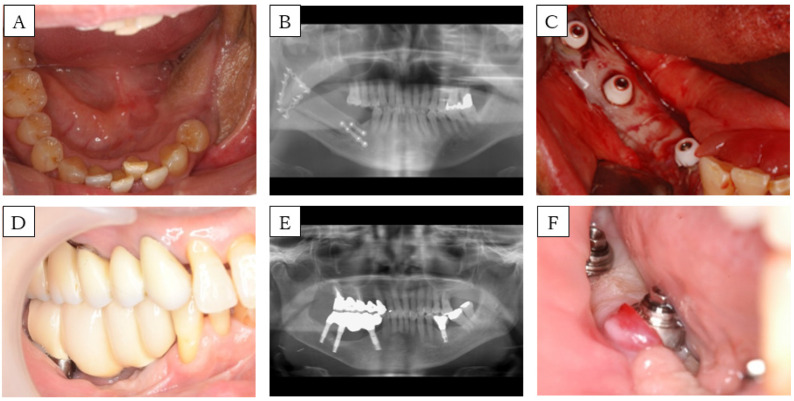
Case 2. (**A**) Preoperative intra-oral picture of the mandibular reconstructions with fibula-free flaps. (**B**) panoramic radiograph of the patient before implant placement. A single-barrel fibula was placed corresponding to the mandible. (**C**) Postoperative intra-oral picture of the FGG. (**D**) Intra-oral picture and (**E**) panoramic radiograph of the patient after setting the prosthesis. (**F**) Intra-oral picture during follow up. Peri-implant mucosa is reddish and swollen.

A 58-year-old woman had an implant-assisted dental rehabilitation planned for oral rehabilitation. She was diagnosed with right lower gingival cancer (T2N1M0), and was treated with segmental mandibulectomy, modified radical neck dissection, and fibula-free flap mandible reconstruction about one year ago (Figure 3A,B). After surgery, we performed rehabilitation with a partial denture, but sufficient mastication was difficult to achieve. First, nail extraction was performed, and split-thickness skin was applied. About one year later, the implant was placed, and the flap was covered. Six months later, FGG was performed concurrently with the secondary implant surgeries. Full thickness dissection is carried out at the site of implant placement. Palatal mucosa was collected, a hole was punched in the part corresponding to the implant with a gingival punch, and the palatal mucosa was placed around the implant. The lower part of the healing cup was cut, the splint was fixed between the abutment and the cup, and COE-PACK^®^ was used to compress and protect the transplanted mucosa (Figure 3C). When the splint was removed four weeks later, the transplanted mucosa was necrotic and fell off. Subsequently, irrigation continued, and scar tissue formed around the implant. Four months later, the prosthesis was set (Figure 3D,E), but infection around the implant was frequently observed (Figure 3F); thus, antibiotics were administrated, and curettage was performed.

### 3.2. Case 3 (Method 2)

**Figure 4:**
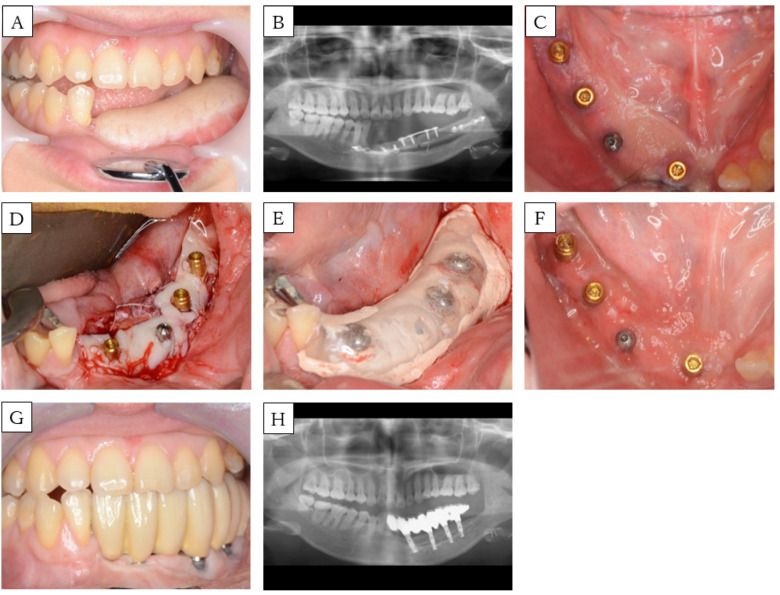
Case 3. (**A**) Preoperative intra-oral picture of the mandibular reconstructions with fibula-free flaps. (**B**) Panoramic radiograph of the patient before implant placement. A single-barrel fibula was placed corresponding to the mandible. (**C**) Postoperative intra-oral picture of the secondary implant surgeries. (**D**) Intra-operative picture: placement of palatal mucosa around the implant. (**E**) Intra-oral picture: a splint is fixed in with a locator abutment in combination with COE-PAK^®^. (**F**) Postoperative intra-oral picture of the FGG. (**G**) Intra-oral picture and (**H**) panoramic radiograph of the patient after setting the prosthesis.

A 30-year-old woman was scheduled for implant-assisted dental rehabilitation for oral rehabilitation. She was diagnosed with mandibular osteosarcoma, and was treated with marginal mandibulectomy and fibula-free flap mandible reconstruction about two years ago (Figure 4A,B). After surgery, we performed rehabilitation with a partial denture, but again, mastication was difficult to achieve. First, we modified the split-thickness skin and formed the alveolar ridge. Approximately one year later, the implant was placed, and another year after that, secondary implant surgery was performed. A bacterial infection was observed around the implant, so antibiotics were administered. In addition, local lavage was performed, and the inflammation was extinguished before FGG (Figure 4C). The flap was formed with a partial thickness flap, the lingual and buccal flaps were fixed to the periosteum of the mandible, and the oral vestibule was expanded. Palatal mucosa was collected, a hole was punched in the part corresponding to the implant with a gingival punch, and the locator abutment was placed (Figure 4D). The wound was covered with COE-PACK^®^, and a splint with a locator cup was attached to protect it (Figure 4E). After three weeks, the splint was removed, and the engraftment of palatal mucosa was confirmed (Figure 4F). Approximately three months after FGG, a fixed prosthesis was set (Figure 4G,H). At present, part of the implant body is exposed because of bone resorption, owing to peri-implantitis that occurred before FGG. Nevertheless, the aesthetic and functionality are good, and no problems have arisen.

### 3.3. Case 5 (Method 3)

**Figure 5:**
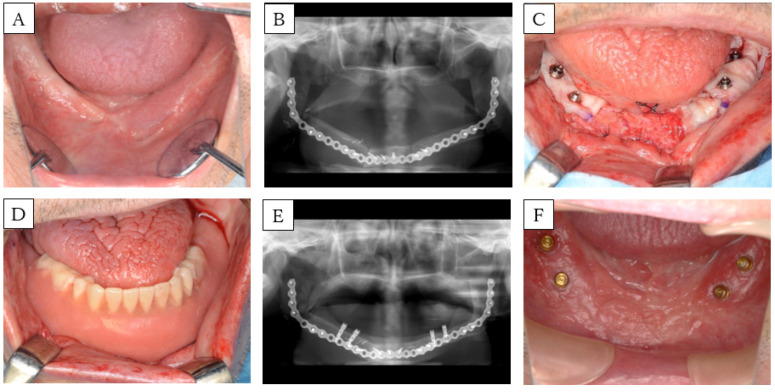
Case 5. (**A**) Preoperative intra-oral picture of the mandibular reconstructions with fibula-free flaps. (**B**) Panoramic radiograph of the patient before implant placement. A single-barrel fibula was placed corresponding to the lower border of the mandible to reconstruct the ablative defect. (**C**) Intra-operative picture: placement of palatal mucosa around the implant. (**D**) Intra-oral picture: rebasing during surgery, installing a temporary abutment, and fixing in dentures using screws. (**E**) Panoramic radiograph and (**F**) intra-oral picture after the FGG.

A 63-year-old man had an implant-assisted dental rehabilitation planned for oral rehabilitation. He was diagnosed with osteoradionecrosis of the jaw, and was treated with segmental mandibulectomy and fibula-free flap mandible reconstruction about one year ago (Figure 5A,B). He had a history of chemoradiotherapy (CDDP+5-FU, Total 65 Gy) for oropharyngeal cancer. First, an implant was placed, and six months later, we applied split-thickness skin and performed FGG. Partial thickness dissection is carried out at the site of implant placement. The flap on the lingual side was fixed to the periosteum of the fibula, the flap on the buccal side was fixed to the periosteum and skin of the fibula, and the oral vestibule was expanded. Denture rebasing was conducted during surgery, in which a temporary abutment was installed. Palatal mucosa was collected, and a hole was punched in the part corresponding to the implant with a gingival punch, and placed around the implant (Figure 5C). Then, the denture was fixed to the implant with a screw (Figure 5D). The inner surface of the denture was relieved by about 1 mm, in consideration of the thickness of the palatal mucosa. The denture was removed after four weeks, the wounded area was irrigated, and the denture was adjusted. The same treatment was conducted weekly, and after one month, transition to a denture was achieved using a locator abutment (Figure 5E,F). Currently, there are no aesthetic or functional problems.

## 4. Results

**Figure 6:**
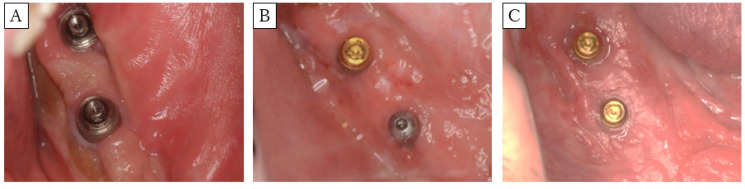
Clinical photograph of postoperative FGG. (**A**): Method 1 (Case 1), (**B**): Method 2 (Case 3), (**C**): Method 3 (Case 5). Method 3 showed the largest (significantly) keratinized mucosa width on both the buccal and lingual sides.

**Figure 7:**
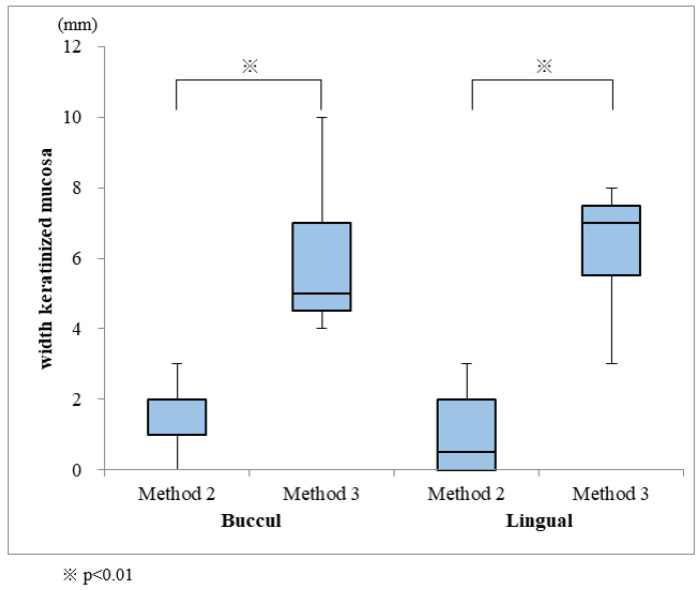
Comparison of keratinized mucosa widths obtained from Methods 2 and 3.

FGGs were performed for 19 of the installed implants; grafts were covered by Method 1 for 3 implants, and by Methods 2 and 3 for 8 implants each (Figure 6). The widths of the keratinized mucosa on the buccal and lingual sides were 5.9 mm and 6.4 mm, respectively, for Method 3, and 1.6 mm and 1.0 mm for Method 2. Keratinized mucosa could not be obtained by Method 1 because the palatal mucosa fell away (Table 2). Method 3 showed a significantly larger keratinized mucosa width on both the buccal and lingual sides, when compared with those measured in Method 2 (Figure 7). One of the two implants was lost in the patient not subjected to FGG.

## 5. Discussion

In this study, among the two implants in the patient on whom an FGG was not performed (Case 1), one implant was lost. Inflammation around the implant was recurrent in the patient for whom the palatal mucosa did not adhere (Case 2). In contrast, the other patients (Cases 3–5) acquired sufficient keratinized mucosa width via FGG, which showed decreased inflammation around the implant compared to that observed in Cases 1–2. These findings suggest that if the soft tissue around the implant is a skin flap, the implant installation for the mandibular reconstruction with fibula-free flap leads to a high risk of inflammation around the implant. When corrective measures to improve the soft tissues were not implemented, poor hygiene around the implants, hyperplastic peri-implant tissue, soft tissue infections and abscesses that start as peri-implant marginal bone loss were all observed, and this may eventually lead to loss of the implant and the dental rehabilitation [2,11]. These results are consistent with the observations reported by Sozzi et al. [5] that suggest the optimal soft tissue around an implant should be immobile, thin, soft, smooth (wrinkle-free) and hairless. Similar to the results of Chang et al. [10], our findings show that the use of FGGs was beneficial in the three methods implemented.

In Case 2 using Method 1, the cine was sandwiched between the improved abutments, and the transplanted palate mucosa was pressed; however, the cine was insufficiently tight, and the marginal exclusion was sufficient. Therefore, it was considered that the transplanted mucosa did not survive. In Case 3 using Method 2, a mandibular fibula reconstruction was performed for reinforcement after marginal resection, and the bone height was sufficient; there were few defects in the oral floor and buccal mucosa, and the required area for transplantation was 1 cm. A good engraftment of the palatal mucosa was obtained because it was buccolingually narrow. In the first case of Case 4, using Method 2, the FGG had a large defect area of 5 × 4 cm^2^, and there was a large loss of tissues on the labial and oral floors. Therefore, sufficient engraftment of the palatal mucosa could not be obtained. In Case 4 (SD-DGER and soft tissue treatment, Method 2) and in Case 5 (a case that required mucosal treatment for the entire lower jaw), the dentures used before the FGG surgery were rebased, and temporary abutments to fix-in the dentures were installed using screws, which was followed by applying pressure to the wounded area to protect it (soft tissue treatment, Method 3). Due to the close contact of the rebased dentures fixed in place with screws, the application of pressure to the wounded area was possible with this method, rendering the dentures stable and resulting in satisfactory exclusion at the borders; this may result in a wider keratinized mucosa, as compared to those observed with other methods. These results suggest that Method 2 can be used for mucosal transplantation at a narrow site; however, Method 3 needs to be performed when extensive treatment is required.

Furthermore, regarding the number of fibulae used for mandibular reconstruction when conducting soft tissue treatment, single-barrel reconstruction leaves a high vertical gap between the fibula bone and the existing bone. This gap leaves room for skin flap thickening. However, it could lead to denture soreness when using a prosthesis made from jaw dentures with a mobile upper structure [2]. Additionally, a comparison of soft tissue treatments conducted in patients undergoing single- and double-barrel reconstruction demonstrated that the latter acquired more keratinized mucosa [2], indicating that double-barrel reconstruction may be more suited to soft tissue treatment around implants. In this study, three patients (Cases 2, 4 and 5) underwent mandibular segmentectomies, and received double-barrel (Cases 2 and 4) or total jaw single-barrel (Case 5) mandible reconstruction; consequently, a comparison could not be conducted. However, the residual bone in the double-barrel reconstruction remained roughly at the same height, indicating its effectiveness as a soft tissue treatment. Fibula-mounting simulations to observe implant treatments may also be important. However, the small sample size was a limitation of this study, and further investigation is necessary. In the future, we plan to conduct a long-term follow-up study on the width of the keratinized mucosa obtained in the tissue around the implants.

## 6. Conclusions

In this study, we successfully compared three soft tissue treatments using FGG for implants after mandibular reconstruction with a fibula-free flap. Our results suggest that an FGG using Method 3 can be a useful soft tissue treatment approach to mandible reconstruction surgeries.

## Figures and Tables

**Table 1:** Patient list.

Case	Age (years)	Sex	Cause	Mandible Defect Size (cm)	Mucosal Defect Size (cm)	No. of Implants	View	FGG	Fixing Method of Mucosal Graft	Prosthesis	Timing * (months)	Implant Lost
1	46	M	SCC	6.5	3 × 2	2	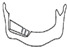	no	-	Fixed	-	1
2	58	F	SCC	5	4.5 × 1.5	3	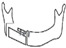	yes **	Method 1	Fixed	0	0
3	30	F	OS	7	5 × 1	4	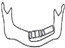	yes	Method 2	Fixed	3	0
4	63	M	SCC	5	5 × 4	4	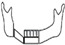	yes	(1) Method 2 *** (2) Method 3	Removable	(1) 7 (2) 31	0
5	66	M	ORN	17	All Gingiva	4	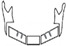	yes	Method 3	Removable	0	0

F: female, M: male, FGG: free gingival graft, SCC: squamous cell carcinoma, OS: osteosarcoma, ORN: osteoradionecrosis; * Period of time following second-stage implant surgery till FGG was conducted; ** The grafted palatal mucosa did not take and fell away; *** A sufficient gingival width could not be deposited—thus, we reconducted the FGG.

**Table 2:** Keratinized mucosa width measurements for Methods 1–3.

		Width of Keratinized Mucosa (mm)					
	n	Buccal			Lingual		
	(p/i)	Mean	Median	SD	Mean	Median	SD
Method 1	1/3	0	0	0	0	0	0
Method 2	2/8	1.6	2	0.9	1	0.5	1.2
Method 3	2/8	5.9	5	2.1	6.4	7	1.9

n = number of patients (p)/implants (i).
